# Emotion-Attention Interaction in the Right Hemisphere

**DOI:** 10.3390/brainsci11081006

**Published:** 2021-07-29

**Authors:** Kaisa M. Hartikainen

**Affiliations:** 1 Behavioral Neurology Research Unit, Tampere University Hospital, 33521 Tampere, Finland; kaisa.hartikainen@live.com or kaisa.hartikainen@tuni.fi; 2 Faculty of Medicine and Health Technology, Tampere University, 33520 Tampere, Finland

**Keywords:** emotion, threat, attention, right hemisphere, ERP, global processing, inhibitory control, frontal alpha asymmetry, FAA, biomarker

## Abstract

Hemispheric asymmetries in affective and cognitive functions have been extensively studied. While both cerebral hemispheres contribute to most affective and cognitive processes, neuroscientific literature and neuropsychological evidence support an overall right hemispheric dominance for emotion, attention and arousal. Emotional stimuli, especially those with survival value such as threat, tend to be prioritized in attentional resource competition. Arousing unpleasant emotional stimuli have prioritized access, especially to right-lateralized attention networks. Interference of task performance may be observed when limited resources are exhausted by task- and emotion-related processing. Tasks that rely on right hemisphere-dependent processing, like attending to the left visual hemifield or global-level visual features, are especially vulnerable to interference due to attention capture by unpleasant emotional stimuli. The aim of this review is to present literature regarding the special role of the right hemisphere in affective and attentional brain processes and their interaction. Furthermore, clinical and technological implications of this interaction will be presented. Initially, the effects of focal right hemisphere lesion or atrophy on emotional functions will be introduced. Neurological right hemisphere syndromes including aprosodia, anosognosia and neglect, which further point to the predominance of the intact right hemisphere in emotion, attention and arousal will be presented. Then there will be a brief review of electrophysiological evidence, as well as evidence from patients with neglect that support attention capture by emotional stimuli in the right hemisphere. Subsequently, experimental work on the interaction of emotion, attention and cognition in the right hemispheres of healthy subjects will be presented. Finally, clinical implications for better understanding and assessment of alterations in emotion–attention interaction due to brain disorder or treatment, such as neuromodulation, that impact affective brain functions will be discussed. It will be suggested that measuring right hemispheric emotion–attention interactions may provide basis for novel biomarkers of brain health. Such biomarkers allow for improved diagnostics in brain damage and disorders and optimized treatments. To conclude, future technological applications will be outlined regarding brain physiology-based measures that reflect engagement of the right hemisphere in affective and attentional processes.

## 1. Introduction

Hemispheric asymmetries have been extensively studied, with vast neuropsychological and neuroimaging evidence pointing to right hemisphere bias in emotional and attentional processes. Emotional and attentional processes are intricately intertwined. Attention allows selection of the most relevant event for enhanced processing at any given moment. According to the biased competition model of selective attention, there is attentional competition for the brain’s limited neural processing resources [1]. Both stimulus-dependent bottom-up, as well as goal-dependent top-down, mechanisms bias attentional competition. Emotional stimuli, especially those with survival relevance such as threat, are prioritized in this attentional competition [2]. A stimulus that gains dominance in one system, such as within the occipital area responsible for early visual processing, will gain similar dominance in other higher order systems, such as frontal and parietal associative areas involved in higher cognitive processes [3]. Survival depends on a robust neural mechanism that directs attention instantly towards objects or spatial locations of threat-related emotional stimuli, allowing adaptive behaviors swiftly to take place to minimize danger. Such robust neural mechanisms underlying the interaction of emotion and attention are likely subserved by close anatomical interconnections within the right hemisphere.

Emotional stimuli capturing attentional resources may result in a temporary cost to other stimuli that rely on the same pool of processing resources [2,4,5,6]. Consequently, interference in ongoing task performance may be observed, especially in those tasks that rely on the right hemispheric processing resources that are prioritized to the threat-related stimuli. While many studies support right hemisphere-lateralized emotional [7] and attentional processes, there are few studies investigating how emotion–attention interactions within the right hemisphere impact other right-lateralized cognitive processes.

To that end, the main aim of this review is to present studies and evidence that provide insight into the right hemisphere-lateralized emotion–attention and emotion–cognition interactions. Furthermore, the aim is to present potential clinical and technological implications of these interactions. The review starts by briefly explaining brain lateralization and hemispheric specialization. Then neuropsychological evidence for the role of the right hemisphere in emotion, attention and arousal will be presented. Specifically, well-known neurological right hemisphere syndromes with deficits in attentional and affective processes and arousal will be introduced. Subsequently, neuropsychological and electrophysiological evidence for the prioritized access of emotional stimuli to the right hemisphere’s attentional networks will be briefly described. Finally, the review aims to illuminate the interaction of emotion, attention and cognition in the right hemisphere by presenting some relevant studies on this interaction. The review concludes with potential clinical and technological implications of assessing emotion–attention interaction in the right hemisphere.

## 2. Brain Lateralization and Hemispheric Specialization

Motor and sensory functions of the brain are undoubtedly lateralized with each cerebral hemisphere responsible for the opposite side of the body. Despite some asymmetries in cognitive functions, they are conducted in a complementary and integrative manner in the two cerebral hemispheres [8]. However, the two hemispheres are thought to be asymmetrically involved in many attentional, cognitive and emotional functions, with the degree of asymmetry varying depending on the particular mental function in question. Lateralization of brain function allows hemispheres to specialize to different mental functions [9]. For example, the left hemisphere is known to be specialized in language, with left hemisphere lesions frequently resulting in aphasia i.e., a deficit in linguistic processes such as comprehension or production of language [10]. While the left hemisphere processes linguistic information that conveys the meaning of the words, the right hemisphere processes prosody, the intonations that convey emotional information of the speech [11].

Language functions, in the majority of the people, are lateralized to the left hemisphere, whereas emotion, especially negative emotion, attention and arousal seem to be lateralized to the right hemisphere. Originally human brain lateralization was considered unique to human species and related to the development of handedness [12] and language. See review by Michael Corballis [8] on the evolution of lateralized brain circuits where he uses the evolution of language, potentially from the mirror-neuron system [13], as an example. However, separate from language development and not unique to humans, negative emotions seem to be lateralized to the right hemisphere in a wide range of species, from reptiles to mammals [14,15].

Lateralization of mental functions and hemispheric specialization may provide basis for optimized emotional and cognitive functioning and contribute to successful social behaviors. In humans, successful social communication relies on many right hemisphere-mediated functions, from understanding and expressing emotion to understanding sarcasm [16], humor, and metaphors. Understanding figurative language, i.e., the intended meaning rather than the literal meaning of words, may be impaired in some psychiatric and neurodevelopmental disorders like schizophrenia [17] and autism spectrum disorders (ASD) [18]. Like many other brain disorders, schizophrenia is thought to be linked with abnormal or reduced brain lateralization accounting for some of these deficits [19,20]. Likewise, alterations in brain lateralization in ASD have been linked with difficulties in social functioning [21]. To that end, atypical brain lateralization may underlie some psychiatric and neurodevelopmental disorders with emotional and social challenges.

## 3. The Right Hemisphere in Emotion, Attention, and Arousal

### 3.1. Right Hemisphere Lesion—Aprosodia and Other Emotional Deficits

Right hemisphere lesions are known to result in deficits in understanding [22,23] and production of prosody [24,25]. A lesion to brain regions in the right hemisphere, that results in motor, sensory, conduction or global aprosodia, mirror those seen in the left hemisphere in different types of aphasia [26]. In other words, the brain regions devoted to comprehension of language (sensory) and production of speech (motor) in the left hemisphere correspond to those in the right hemisphere devoted to understanding and producing prosody. However, deficits in emotional processing due to the right hemisphere lesion are not limited to prosody but include a broad range of emotional functions including perception and evaluation of emotional expression [27,28] and understanding the emotional meaning of sentences depicting facial, prosodic or gestural expressions [29]. Subjects with right hemisphere lesions have difficulty in emotional perception across different channels; facial, prosodic and lexical [30].

In addition to patients with focal right hemisphere lesions [31], patients with neurodegenerative brain disorders, such as frontotemporal dementia (FTD) with asymmetric brain atrophy, point towards the right hemisphere’s critical role in emotion [32,33]. Patients with FTD show impaired emotion comprehension, especially negative emotion, with greater impairment linked with greater atrophy in the right amygdala and the right orbitofrontal cortex (OFC) [32]. Normally identification of facial emotional expressions seems to be linked with automatic imitation of facial expressions. In the right temporal variant of FTD, automatic imitation is reduced and the coupling between imitation and identification is lost [34]. Emotional blunting is frequently observed in FTD [35,36], for example reactions to disgusting videoclips are reduced [37].

In monkeys emotional blunting has been reported by Murray et al. after unilateral OFC and amygdala lesions [38]. In their study, emotional blunting did not differ between left- and right-sided lesions. In humans, emotional blunting and depression have been reported, for example, after a left basal ganglia stroke [39], but according to a meta-analysis, apathy and depression were not linked to laterality of the lesion [40]. In the study by Murray et al. [38] in monkeys, emotional blunting was observed to stimuli that resemble biologically relevant threat-related stimuli with survival value (rubber snakes) but not to socially threatening emotional stimuli (unfamiliar human intruders). To that end, it is clear, that distinct brain regions mediate responses to emotional stimuli with survival value than those with social relevance, even if both convey threat.

### 3.2. Right Hemisphere Lesion—Anosognosia and Neglect

Right hemisphere lesions are known by neurologists since the famous Babinski [41], to sometimes result in anosognosia, a curious unawareness of neurological deficits. Sometimes patients with anosognosia are unaware of even severe deficits, such as hemiplegia, and are emotionally indifferent [31]. Anosognosia is also seen in neurodegenerative disorders, such as FTD. Atrophy of the right frontal region is involved in loss of awareness, especially concerning those alterations of personality or behavior that are linked with emotion [42]. Another frequent concomitant of a right hemisphere lesion that frequently co-occurs with anosognosia is hemispatial neglect, the inability to attend to and be aware of objects and events in the left hemispace.

Hemispatial neglect is far more frequent after right hemisphere lesion than left hemisphere lesion. This is probably due to the right hemispheric attentional functions spanning across both hemispaces, unlike those of the left hemisphere that span only over the contra-lateral right hemispace [43,44]. Thus, while attention to the right hemispace maybe mediated by either left or right hemispheres, attention to the left hemispace relies only on the right hemisphere. Hemispatial neglect due to right hemisphere lesion highlights the special role of the right hemisphere in attention-related processes [43].

### 3.3. Right Hemisphere Lesion—Hypoarousal

In addition to predominance in attention there is evidence pointing to the right hemisphere dominance in arousal. Neglect may be accompanied with emotional indifference and hypoarousal, highlighting a special role of the right hemisphere not only in attention and emotion but also in arousal [45]. Lesions in the right hemisphere have been reported to result in hypoarousal, reflected in autonomic measures such as galvanic skin response (GSR), in response to alerting or emotional stimuli [45,46,47,48,49]. On the other hand, widespread right hemisphere activations have been observed in the context of sexual arousal [50]. Greater autonomic responses to negative emotional stimuli have been observed when presented to the right hemisphere in comparison to the left hemisphere [51]. There are also hemispheric asymmetries in emotional conditioning, with right hemisphere bias reflected in GSR to conditioned stimuli [52,53]. Interesting evidence supporting right hemisphere’s role in emotional arousal especially in response to aversive stimuli comes from subjects with complete agenesis of the corpus callosum [54]. These subjects show reduced verbal rating of arousal related to aversive stimuli, despite GSR evoked by these stimuli. It seems that while the right hemisphere reacts to aversive stimuli by emotional arousal, the left hemisphere, which is responsible for giving the verbal report on arousal, was not fully aware of this response. The missing corpus callosum resulted in lack of communication between the two hemispheres.

Arousal and attention are intricately intertwined, with an appropriate level of arousal required for successful attentional processes. Physiological arousal varies from moment to moment and is affected by numerous internal and external factors, including emotional stimuli and especially threat-related stimuli. Arousal enhances attention to goal-relevant salient information, while suppressing attention to other information, in young healthy subjects [55]. Efficient functional connectivity of the locus coeruleus and frontoparietal networks provides a functional link between arousal and attention networks. In older adults, functional connectivity of arousal and attention networks declines, and arousal leads to increased processing of both salient and non-salient visual information. This predisposes to increased susceptibility to distraction. The impact of arousal on attention is likely dependent on the level of arousal, according to the Yerkes–Dodson law [56]. Arousal, along with right hemisphere-lateralized inter-related cognitive functions including attention, awareness and response to novelty, have been suggested as potential mediators for cognitive reserves that influence cognitive outcomes in different brain pathologies, such as Alzheimer’s disease [57]. To that end, a well-preserved right hemisphere is important in healthy aging and alterations in right hemispheric function are involved in aging-related cognitive changes.

In summary, evidence from patients with lesions to the right hemisphere, as well as other neuroscientific literature, supports the idea that the right hemisphere has a special role in emotion, attention and arousal.

## 4. Attention Capture by Negative Emotional Stimuli in the Right Hemisphere

Right hemisphere-lateralized ventral attention system, including the temporoparietal junction (TPJ), is thought to be involved in shifting attention to salient stimuli and targets [58,59,60]. Emotional stimuli are inherently salient and thus capable of attentional capture in the right hemisphere’s attention system. This is especially true for fear or threat-related stimuli that have survival relevance, such as spiders or snakes [61,62]. However, the TPJ is also activated by positive emotions [63] and is thought to have a role in emotional arousal linked with positive emotions [64]. In addition to reorienting attention to salient stimuli within one person’s brain, the right TPJ (rTPJ) is also thought to underlie synchronization of activity between brains of two people and to allow for their shared attention [65,66]. rTPJ has been suggested to play a critical role in various aspects of social cognitive processes, including those underlying theory of mind and empathy [66]. Furthermore, rTPJ has a role in subjective emotional experience with dimensions of different emotions encoded in overlapping gradients [63].

Moreover, rTPJ has been suggested to be a central hub for interpersonal emotional communication, especially important in early development of non-verbal emotional communication and interaction between a caregiver and an infant [64]. This caregiver–infant pre-verbal emotional communication, that relies on prosodic, gestural and facial emotional expressions, provides a basis for the development of attachment. To that end, rTPJ seems also to be involved in the development of attachment. Attachment supports survival at the age when the infant’s survival is entirely dependent on the caregiver. Furthermore, secure attachment allows infant to seek and find security in a caregiver in the face of threat [67]. Carefully monitoring infant’s emotional arousal, the caregiver downregulates negative emotional states of the infant with appropriate and sensitive emotional interaction. Successful emotional communication and downregulation of infant’s negative emotions relies on the right-hemispheric functions of both the caregiver and the infant. This allows the experience-based maturation of an infant’s emotional and social functions and their underlying brain regions and networks, [68] including the rTPJ and rOFC. OFC is known to be involved in downregulating emotions as well as allocating attention to emotion [69,70] with distinct impact of the left and the right OFC on attention-related brain physiology [71]. Any signal indicating threat to bonding with the caregiver has strong threat-related survival value for the infant. Thus, the rTPJ and rOFC may well be key hubs supporting threat detection that relies on the processing of non-verbal emotional information in the right hemisphere. This emotion system is not limited to threat-detection, but extends to positive emotions that support bonding, social connections, health and psychological safety, which also convey the absence of threats to survival. While negative emotions may support survival by promoting rapid and specific actions that allow minimizing danger, positive emotions may promote survival by increasing cognitive-, social- and health-related resources needed to successfully face later, but inevitable, threat [72]. Robust right-lateralized emotion networks that allow efficient capture of attention by emotional stimuli and consequent emotional arousal may have emerged in the evolution to support the survival of the species and further evolved during animal or child development to also support the survival of an individual.

### 4.1. Electrophysiological Evidence

While emotional visual stimuli activate many regions in the distributed networks of both hemispheres [73], there are reports supporting greater right-sided activation by emotionally arousing stimuli [74,75]. Recent fMRI and PET literature will not be reviewed here, but according to some meta-analysis it does not provide support for the right hemisphere dominance in emotion, such as one by Fusari-Poli et al. on laterality of processing emotional faces [76]. In a recent review Gainotti pointed out some of the challenges of emotion research using aforementioned brain imaging methods and called for caution in consideration of the results [7]. For example, the scanner provides a sub-optimal environment for emotion research, limiting full-blown emotional processes. In addition, most imaging studies focus on the cognitive aspects of affective processes rather than emotional, physiological, behavioral or autonomic aspects of affective functions evolving in time [7]. Moreover, the right hemisphere has been shown to react faster than the left hemisphere to emotional events [77] and the right amygdala to habituate faster than the left [78]. Temporal differences in affective processes of the two hemispheres are lost using fMRI and PET but may well be captured with EEG and event-related potentials (ERPs) with higher temporal resolution. For example, affective judgements (liking or disliking) of faces modulated ERP responses earliest, at 80 ms, when presented to the right hemisphere (in the left visual field, LVF) in contrast to 104 ms when presented to the left hemisphere (right visual field, RVF) [77]. Differences in temporal resolution may underlie some of the discrepancy between results obtained with different methods of investigation. While fMRI literature is not reviewed here, two early fMRI studies with right-lateralized activations due to emotional stimuli are, however, mentioned. In 1998, Lang et al. [74] reported right-lateralized activation of occipital region due to emotional pictures and a study by Canli et al. [75] published in the same year showed right-lateralized activation due to emotionally negative pictures in frontal regions including right gyrus rectus of the OFC.

Many electrophysiological studies support increased attentional allocation to and enhanced processing of emotional stimuli in the early and later processing stages, especially in the right hemisphere. Electrophysiological studies by Keil et al. report right-lateralized emotional effects in ERPs, in late gamma band [79], and in steady-state visual evoked potentials (SSVEPs) [80,81]. Emotional stimuli evoke larger P1, N1, P3, N2-P3 and late positive potential (LPP) amplitudes than neutral stimuli [79,82,83] with many of these effects lateralized to the right hemisphere [4,79,80,81,83]. 

Further evidence for the right hemisphere attention capture by emotional visual stimuli comes from diminished N2-P3 ERP complex to targets presented to the right hemisphere (LVF), when preceded by affective stimuli [4]. N2-P3 peak-to-peak amplitude is thought to reflect attention allocation as it increases with behaviors reflecting greater attention [84]. To that end, these results suggest that processing of emotional stimuli is prioritized over LVF targets that compete for the same pool of right hemisphere attentional resources.

N2-P3 is also larger for threat-related than neutral stimuli in healthy subjects with enhanced effect of emotion in mild traumatic brain injury (MTBI) [85]. While top-down control functions limit automatic bottom-up influences of emotional stimuli in healthy subjects these control functions may be compromised in MTBI leading to greater attention capture by unpleasant emotional stimuli.

We have reported right-lateralized effect for emotional enhancement of N2-P3 in healthy subjects, while a similar effect in patients with OFC lesion was lacking [83]. OFC lesion led to alterations in emotional responses and their dynamics pointing to the role of OFC in normal emotion–attention interaction [69]. N2-P3 complex is followed by LPP and thought to reflect sustained motivated attention [86]. LPP is enhanced by emotional stimuli, with a right-lateralized effect for faces perceived as positive or negative [87].

### 4.2. Emotional Stimuli Overcome Neglect

In addition to electrophysiological data, evidence from neurological patients support automatic attention capture by emotional stimuli in the right hemisphere. Biologically relevant threat-related stimuli can overcome attentional deficits in patients with visuo-spatial neglect. Patients with neglect were more frequently able to detect line-drawings of spiders in their neglected left hemifield than emotionally neutral control images resembling flowers [88]. Similar phenomena has been observed with emotionally negative complex visual scenes [89]. In addition, faces with emotional expression were faster detected than faces with neutral expression in a visual search task by patients with neglect even in their neglected hemifield [90]. This emotional advantage was partly modulated by the OFC. There was a smaller beneficial effect of emotion in those patients with lesion to the OFC. The OFC has an important role in allocation of attention to emotional stimuli [69] as well as modulating negative emotional responses [91]. To conclude, when OFC is intact, patients with neglect benefit from emotional stimuli in overcoming inattention to stimuli in their left contralesional neglected hemifield.

Emotional stimuli may lead to increased activity in the right amygdala even before conscious detection [92]. Detection is dependent on a longer and slower cortical pathway, but a rapid subcortical route for emotional stimuli [93,94] may influence arousal even before detection. The right amygdala is thought to be especially involved in unconscious processing of threat-related emotional stimuli via the rapid subcortical route [7,92,93]. Stimulus-evoked arousal is linked with locus coeruleus-mediated (LC) norepinephrine (NE) activation, thought to favor perception of salient high-priority representations [95]. Increased arousal is known to improve detection of objects in the neglected hemifield of patients with hemispatial neglect.

### 4.3. Emotional Stimuli May Cause a Neglect-Like Phenomena in Healthy Subjects

While in some contexts neglect may be mitigated, the opposite happens in healthy subjects with low arousal due to sleep-deprivation [96]. Sleep-deprivation results in a neglect-like phenomena with slowed reaction times (RTs) to invalidly cued stimuli in the left hemifield. A neglect-like phenomena in healthy subjects may also occur temporarily due to emotional stimuli. Inattention to the left hemifield may occur due to unpleasant emotional stimuli automatically capturing right hemispheric attentional resources [2,4]. Similarly motivationally salient stimuli capture right hemisphere processing resources and interfere with visual search in the LVF [97]. Neglect-like phenomena in healthy subjects has been thought to be associated with right hemisphere-dominant brain functions leading to subtle imbalances in the attentional functions between the left and right visual fields or hemispaces. fMRI study in healthy subjects has shown that perceptual pseudoneglect is associated with the bilateral—yet right-dominant—attentional orienting system that includes brain areas, such as the frontal eye field (FEF) [98], which damage leads to an actual neglect.

## 5. Attentional Competition in the Right Hemisphere

The limited and, to some extent, independent pool of neural processing resources within a hemisphere [99,100] may lead to competitive interactions between stimuli and tasks that are predominantly processed within the same hemisphere. When competition occurs for the same resources within one hemisphere as opposed to between hemispheres greater behavioral, perceptual or electrophysiological interference is expected [4]. Emotional stimuli, even when unattended, tend to be favored in attentional resource competition in the right hemisphere at the expense of task-relevant stimuli, leading to interference in task performance [2,4].

More than two decades ago we reported that subjects were slower to detect targets in the left hemifield when preceded by emotional stimuli, either pleasant or unpleasant, in comparison to neutral stimuli [2]. The slowing of reaction times (RTs) was greatest in context of unpleasant emotional stimuli. The results suggested an automatic attention capture by emotional stimuli and interference of right hemispheric function, i.e., attending to the left hemifield, especially by unpleasant stimuli. Subsequently, we reported reduced N2-P3 amplitude to targets presented to the right hemisphere (LVF), when preceded by emotional stimuli [4]. The reduction was greatest to unpleasant stimuli and in the right parieto-temporal region. Thus, in addition to behavioral evidence, electrophysiological evidence supports attention capture by emotional stimuli in the right hemisphere and, consequently, reduced attentional allocation to the LVF targets; right hemisphere-dependent attentional processes were temporarily compromised due to attention capture by emotional stimuli. Correspondingly, priming by motivationally salient distractors has been reported to slow visual search in the LVF, but not in the RVF, suggesting that motivational stimuli, similar to emotional stimuli, compete for control with non-emotional cognitive processes in the right hemisphere [97].

### 5.1. Competition between Negative Emotional Stimuli and Global Visual Stimuli

As we had previously discovered that emotional stimuli interfere with attention to the LVF [2,4], we wanted to further assess whether other right hemisphere-dependent functions are also vulnerable to temporary interference due to attention capture by emotional stimuli. To that end, we investigated the impact of emotional stimuli on global and local levels of visual processing. Processing global-level visual features has been suggested to rely predominantly on the right hemisphere, while processing local-level visual features on the left hemisphere [101]. Alternatively, the right hemisphere has been suggested to be more adept at low-frequency information processing and left hemisphere at high-frequency processing [102]. For example, the right hemisphere conducts complementary speech processing on mainly low-frequency information in the auditory signal [103]. This contrasts with the higher frequency auditory information content processed predominantly by the language-dominant left hemisphere. Similar laterality effects have been detected in both auditory and visual perception, suggesting a general computational difference between the two hemispheres. In other words, left hemisphere computation is biased for high frequency processing while right hemisphere computation for low-frequency processing [104].

To that end, we expected greater interference with global- (right hemisphere) than local- (left hemisphere) level visual processing, due to unpleasant emotional stimuli, that readily capture right hemisphere attentional resources [6]. We assessed the impact of preceding negative emotional stimuli on detection of subsequent global- and local-level visual targets. Targets were Navon type hierarchical letters [105], composed of small local letters forming a larger global letter. Indeed, when presented with negative emotional stimuli, subjects made more errors in detecting subsequent global level visual targets. There was also a reduction of global target detection-related ERPs, specifically N2-P3 peak-to-peak amplitude, over the right parietal region in the context of preceding unpleasant emotional stimuli. This finding resembles the impact of unpleasant emotional stimuli on subsequent LVF target detection ERPs, with reduction in N2-P3 amplitude [4]. Reduced attention allocation to global level targets was observed due to preceding unpleasant emotional stimuli. In conclusion, unpleasant emotional stimuli were shown to interfere with right hemisphere-dependent global-level processes. These results suggest there was an attentional resource competition within the right hemisphere between unpleasant emotional stimuli and global level visual stimuli, favoring emotional stimuli. Such competition did not occur in the left hemisphere as there was no interference with the left hemisphere-dependent local-level processing due to either negative or positive emotional stimuli. These findings, linking negative emotional stimuli with interference in global processing, are in line with other studies linking negative mood and depression with reduction in the tendency to attend to the bigger picture and prioritize global level processing [106,107]. Correspondingly, in healthy subjects, hearing a suspenseful narrative suppressed attention to the visual periphery, narrowing attentional focus [108].

Young healthy subjects have a global perceptual bias i.e., tendency to see the big picture over the details, unlike older subjects [109], subjects with depression [106], attention deficit hyperactivity disorder (ADHD) [110] or autism spectrum disorder [111]. Aging, negative mood and neurodevelopmental disorders interfere with a normal tendency to see the big picture over the details. Thus, it is of importance to enforce positive emotional environments to shield global level processing from emotional interference in older subject and clinical populations. Negative emotional stimuli and mood may bias local- over global-level processing [6], and, for positive emotions, global over local processing; in other words, negative emotions tend to narrow the scope of attention and positive ones broaden it [112]. There may be a link the other way around, as well; engaging in a task related to global, in contrast to local, processing was shown to support positive mood while viewing a sad movie [113]. Future studies are needed to shed more light on the mechanisms of emotion–cognition interaction. It remains to be studied whether engaging in global processing promotes a broader mindset and subsequently positive mood or whether active engagement of the right hemisphere in global processing interferes with processing negative emotional information and consequently shields from negative mood. Better understanding of these interactions have implications for promoting efficient and healthy working environments that support positive mood, cognition and brain health in general. In addition, rehabilitation of brain damage and therapy for mood disorders may benefit from better understanding of emotion–cognition interactions in the right hemisphere. As an example to that end, a recent study highlights the importance of rTPJ in counselor–client brain synchronization during psychological counselling [114].

### 5.2. Interaction between Negative Emotional Stimuli and Response Inhibition

In addition to perceptual level competition for processing resources between emotional stimuli and task-relevant stimuli there is a competition at higher processing levels, including executive control [115]. High-threat stimuli activate wide cortical and subcortical networks and regions and are thought to consume resources related to attentional and effortful control. An unexpected threat, outside the scope of a current task, calls upon executive functions to minimize it. In addition, inhibitory control functions are needed for inhibiting undue emotional reactions and inappropriate behavioral responses. Inhibitory control of motor and emotional responses are both thought to engage right-lateralized networks [116]. Competition for the shared inhibitory control resources between response inhibition tasks and threat-related inhibitory processes might be expected. If threat-related processing is prioritized, competition for shared resources could lead to a compromised response inhibition performance. This would be in line with previously discussed interference of other right hemispheric functions, such as attending to the LVF [2,4] and global-level visual processing [6], due to threat.

Indeed, this is what we and others have shown. Our team has shown that a task-irrelevant, threat-related simple visual stimuli, such as a line-drawing of a spider, impaired response inhibition accuracy in healthy young subjects [5]. Unlike emotional stimuli frequently used in emotion research, such as the international affective picture system (IAPS) that contains pleasant, unpleasant and neutral photos of events, objects, people or scenes [117], these emotional and neutral images were constructed of identical line elements allowing for control over the low-level visual features, thereby isolating the impact of emotion on response inhibition. Task-relevant images of spiders and snakes [118] and task-relevant fearful [119] and angry faces [120] have been shown to impair response inhibition. On the other hand, negative emotional stimuli may also facilitate the right hemisphere processing and processes related to executive function [121]. Whether facilitation or interference occur depends on several factors, such as the timing of the stimuli and processing load, that determine whether competition for resources will take place. In a study by Choi & Cho, the threat of an electric shock facilitated response inhibition, especially in participants with high state anxiety [122].

Evidence from patients with a lesion to the right hemisphere as well as functional imaging literature point to a special role of the right hemisphere in attentional and inhibitory control functions, which constitute an integral part of executive function [116]. Evidence from patients with lesion to the right frontal pole implicated the right inferior frontal gyrus (rIFG) in inhibitory control [123]. In a review by Aron et al., this inhibitory control was described as a brake function that can either suppress, pause or stop a response or a response tendency, and which may be vulnerable to malfunction in neurodevelopmental disorders, for example attention deficit disorder, causing a wide variety of inhibitory deficits [124]. Further lesion evidence from a split-brain patient indicated that while the left hemisphere is also capable of response inhibition, the right hemisphere response inhibition performance is superior to it [125,126]. While fMRI activations associated with response inhibition depend on a specific task, the right-lateralized network is involved in successful Nogo responses [127]. Based on imaging evidence, the rIFG has been suggested not to be specifically recruited in response inhibition, but rather whenever a salient or task-relevant cue is presented, independent of whether response inhibition is required or not [128]. Negative emotional pictures have been shown also to activate rIFG [75]. fMRI literature points towards IFG’s role in emotional inhibition, as downregulation of emotion leads to increased activity in IFG and decreased activity in amygdala bilaterally [129]. rIFG seems to have a wider role in inhibitory control, executive control of attention and in executive control in general beyond response inhibition [116]. The executive control of attention allows for the selection and prioritization of goal-relevant information along with increased inhibition of irrelevant information. The right hemisphere has been suggested to be superior in the executive control of attention, especially when alerting and orienting functions are needed [130]. rIFG is a potential candidate region, along with its networks, for shared inhibitory control resources needed for both emotional and behavioral control [116]. Reduced activation of rIFG and reduced connectivity between rIFG and other frontal regions has been reported during stopping when preceded by viewing negative images [131].

While attentional and inhibitory control functions in the right hemisphere may be superior to those of the left hemisphere they seem to be vulnerable to interference from unpleasant emotional distractors [2,4,5]. To that end, emotionally arousing pictures have been shown to interrupt ongoing controlled activities, for example response inhibition [5,132,133]. It remains to be studied whether threat predisposing to impulsive behaviors is due to a common resource pool for effortful control being prioritized to deal with threat, especially in the right hemisphere.

The clinical relevance of understanding how negative emotions interact with and possibly impair inhibitory control of behavior relate to neuropsychiatric conditions with challenges in impulse control, such as ADHD, and extend to many behaviors in psychiatric conditions ranging from substance use in addiction [134,135] to impulsive suicide attempts [136]. Impulsive behaviors that negative emotions may predispose to may sometimes have devastating consequences in everyday life even for healthy individuals. The fact that threat-related distractors may increase impulsive responding by interfering with inhibitory control of behavior is important to consider in treatment, rehabilitation and psychoeducation aiming to improve self-management in a wide range of neuropsychiatric populations. In addition, the consequences of the above-described emotion–executive function interaction should be carefully considered and actions to counteract unfavorable consequences should be taken in many work environments, where impulsive responding to negative emotional events might lead to catastrophic outcomes.

### 5.3. Interference of Task Performance Due to Emotional Distractors

While this review focuses on attentional competition as a potential mechanism underlying interference in task performance due to emotional stimuli, there are other mechanisms by which emotional stimuli may either interfere or facilitate cognition, for example freezing, arousal and priming. Whether or not attentional competition between distracting emotional stimuli and task-relevant stimuli occurs depends on several individual-, task-, emotion-, and timing-related factors. Lavie’s perceptual load theory posits that distractor interference is determined by perceptual load, with low-load leading to interference [137]. Along the lines of perceptual load theory [137], it has been suggested that emotional distractors are only processed when the task involved does not capture all processing resources [138]. In other words, if the task is low-demand and attentional resources are available, emotional distractors are processed. In line with results indicating low load tasks allow processing of emotional distractors, we have shown that low attentional engagement in a task makes attention network activity susceptible to emotional interference, especially in the right hemisphere [139]. Thus, in addition to the task difficulty, the level of engagement in a task may have a role on whether emotional stimuli are prioritized over task relevant stimuli. See review by Taylor and Fragopanagos for further review on factors contributing to emotion–attention interaction as well as a proposed neural model underlying this interaction [140].

However, the notion that emotional distractors would only be processed in low-load situations is in contrast to most of our other studies, where subjects are engaged in a cognitively challenging task and yet processing emotional stimuli is prioritized at the expense of task performance [2,4,5,6]. Likewise, in a study by D’Andrea-Penna et al. [141] the greatest interference by emotional stimuli was detected only at the highest level of task difficulty.

It is not surprising that the interference occurs predominantly at high task loads. No interference would be expected when adequate processing resources are available for both task related processes and emotion related processes. Furthermore, it makes evolutionary sense that emotional stimuli indicating potential threat are prioritized independent of the task engagement or difficulty. Potential threat needs to be assessed always and appropriate actions taken to avoid threat, even when busy in a challenging task. It may well be that when engaged in a challenging task, attention is prioritized only to highly arousing negative or threat-related stimuli with survival value [2,4,5,88], but not, for example, social stimuli such as facial expressions [138].

In conclusion, there seems to be attentional competition between negative emotional stimuli and attentional and cognitive processes lateralized to the right hemisphere, such as attention to the LVF, processing of global level visual information and inhibiting prepotent responses. With threat-related emotional stimuli prioritized in this competition, these right hemispheric functions are temporarily compromised. Objective assessment of the right hemisphere-lateralized interaction of threat, attention and inhibitory control may provide a novel window into brain disorders with deficits in these functions or alterations in their interplay. Furthermore, assessing emotional interference of cognitive performance may provide biomarkers for brain disorders and their treatments.

## 6. Clinical Applications

### 6.1. Task-Induced Frontal Alpha Asymmetry (FAA)

Measuring electroencephalography (EEG) provides an objective online tool for investigating fine alterations in brain physiology underlying altered emotion–attention interaction. An EEG-derived measure, frontal alpha asymmetry (FAA), reflects relative activation of each anterior hemisphere. FAA measured during a cognitive task and in context of threat-related distractors depicts subtle alterations in right hemispheric emotion–attention interaction. Consequently, it has potential as a biomarker for affective brain functions. Such biomarkers have shown potential applicability for assessing the impact of neuromodulation [142] and the impact of MTBI on affective neural circuits and functions [143].

In addition to providing a potential biomarker for emotion–attention interaction, FAA literature provides another line of evidence for the right hemisphere dominance in negative emotion, especially those related to withdrawal behaviors [144,145]. FAA is typically derived by subtracting alpha power at the left frontal region (F3 electrode) from that of the right frontal region (F4 electrode). Alpha power is thought to reflect the idle or inhibited state of the cortex, and thus has an inverse relationship with cortical activation, with less alpha meaning greater activation. To that end, negative FAA values indicate relatively greater right frontal activation, typically linked with negative emotional state [144], withdrawal behaviors [146], depression or vulnerability to depression [126]. While FAA has been frequently linked with depression and anxiety, recent meta-analysis found resting state FAA to have limited diagnostic value in depression [147].

### 6.2. Emotional Modulation of FAA—A Novel Biomarker for RH Emotion-Attention Interaction

As resting state FAA has so far failed to be a reliable clinical biomarker of depression [147], we have developed a novel biomarker, based on task-induced FAA, in threatening contexts [142,143]. Engaging frontal networks with a task involving executive functions and right hemispheric networks with negative emotional stimuli allows the assessment of FAA, when competition between an executive function task and emotion-related processes occurs and emotional control is required. This scheme provides a setting where subtle alterations in emotion–attention interaction and emotional control are likely reflected in FAA, unlike during resting state FAA measurements. During active engagement of the frontal circuits, we have shown that emotional modulation of FAA (eFAA) correlates with emotionally modulated behaviors [142] and depressive symptoms [143]. This novel biomarker, eFAA, could overcome some of the weaknesses of the resting state FAA and be a more reliable biomarker of alteration in emotion–attention interaction, extending beyond depression [142,143].

Cognitively engaging tasks leave no room for mind wondering that can introduce uncontrolled factors and cover some of the clinically relevant alterations in FAA. To tap into affective circuits, they need to be activated. Alterations in affective functions, in a clinical population or due to a treatment, need to be assessed during a task that involves a salient emotional component. To that end, we have created a task, the Executive Reaction Time Test, which simultaneously engages several executive functions [148] in the context of task-irrelevant, but biologically relevant, threat-related distractors. The task requires controlled attention, response inhibition, working memory and suppression of undue impact of emotional distractors on task performance. Measuring task-induced FAA during such a task ensures active engagement of the fronto-thalamic and the limbic networks as well as the right-lateralized emotion–attention networks.

Many different brain disorders are linked with atypical asymmetry in activation of frontal cortico-subcortical circuits. Deficits in affective and cognitive brain functions and alterations in emotion–attention interaction are seen in psychiatric, neurodegenerative and neurodevelopmental disorders. Abnormal brain lateralization has been reported, for example, in schizophrenia [19,20], borderline personality disorder (BPD) [149], FTD [33] and ADHD [150], and is thought to account for some of the cognitive and affective deficits and social challenges linked with these disorders. Hypo- or hyperactivity or alteration in normal activity balance between the two hemispheres is reflected in FAA, and alteration in emotion–attention interaction likely reflected in the novel eFAA index. To that end, such a novel biomarker may provide tools for further scientific discovery related to better understanding brain disorders with altered hemispheric lateralization and affective symptoms. The potential clinical applicability of eFAA and other FAA-derived biomarkers of emotion–attention interaction is vast, however more research is needed before these biomarkers provide clinical tools for improved diagnostics, optimized neuromodulation and other treatments, or tools for clinical intervention studies aiming at optimized brain health.

We have previously assessed FAA in patients with refractory epilepsy treated with vagus nerve stimulation (VNS) [151] and deep brain stimulation (DBS) targeted at anterior nucleus of thalamus (ANT) [142]. We showed that shutting off ANT, a key node of the limbic circuitry, with high frequency electric stimulation, had an immediate effect on FAA. Alteration in eFAA was linked with alteration in emotion modulated behavior [142]. This finding supports the idea that task-induced FAA in context of threat-related stimuli reflects the functioning of limbic circuits essential in many of the affective functions. To that end, eFAA has potential as a biomarker for the effect of neuromodulation on the brain’s affective circuitry, allowing immediate assessment of the impact of neuromodulation on individual’s affective brain functions [142].

Objective assessment of the impact of neuromodulation on affective brain functions is especially called for in treatment of mood disorders such as depression, where affective functions are the target of the treatment [152]. Also, when the side effects of neuromodulation involve affective functions, their objective assessment is needed. For example, in treatment of refractory epilepsy with DBS, depressive symptoms may be a side effect [153]. With a huge parameter space that needs to be individually optimized in many neuromodulation treatments and lack of current objective measures of affective brain functions, such biomarkers have important clinical significance in providing immediate objective feedback for such optimization.

## 7. Technological Applications

The applicability of brain physiology-based measures, such as eFAA, reflecting the engagement of the right hemisphere in affective and attentional processes is not limited to the clinical and neuroscientific fields but extends to the field of technology. Biomarkers that are based on measuring especially the right hemisphere’s engagement in a task with emotional content, such as emotional modulation of FAA [143] and threat-modulation of executive function [12], may be used to asses not only clinical populations, such as head injury and depression, but also healthy subjects in interaction with novel technologies. For example, FAA derived measures may be used to study the impact of humanoid robots’ emotional behaviors on human user’s emotional responses. In a recent study negative FAA scores reflecting greater right hemisphere activity and more negative emotional state in a user was observed in response to robot’s sad behaviors [154]. An opposite pattern, with most positive FAA values along with positive emotional state in the user, was observed in response to robots’ joyful behaviors. Thus, by allowing objective and reliable measurement of emotional responses in users, FAA and other biomarkers of affective functions have implications for designing humanoid robots that better mimic humans in their social and emotional behaviors. Furthermore, humanoid robots could be designed to regulate users’ emotional states, with applicability in elderly homes or even as a tool for future therapies in mood disorders.

In addition, assessment of user experience of other novel technologies including self-driving cars [155] may build on measuring, online, the right hemisphere-dependent emotion-related activity of the user. Such human brain physiology-based measures allow for objective assessment of attentional and affective brain functions underlying user experience and provide bases for technological development towards emotionally pleasant user experience.

Finally, eFAA and other brain physiology-based measures reflecting emotional state and alteration in emotion–attention interaction have potential applicability in detecting work-related emotional stress and may provide tools to assess and modify work environments in a way that allow for optimized occupational brain health.

## 8. Conclusions

The reviewed evidence points to the right hemisphere’s special role in attention and emotion. While the review mainly focused on unpleasant emotional stimuli, emotional stimuli, independent of valence, are capable of capturing right hemispheric processing resources [2,4]. Unpleasant threat-related emotional stimuli seem to be especially prioritized in right hemispheric attentional competition. Competition for neural processing resources occurs both at the lower perceptual level, in early sensory cortices [1,100], and at later level of cognitive representations and working memory in the fronto-parietal associative cortices and networks [99]. Assessing the competitive interaction of unpleasant emotional stimuli and tasks that engage the right hemisphere-dependent attentional and cognitive functions [4,6] may allow novel insights into normal emotion–attention interaction in healthy subjects [139].

Understanding the normal interaction of emotion with attention, perception and executive functions in the right hemisphere is crucial for better understanding the alterations in these interactions in a wide variety of neuropsychiatric, neurodevelopmental and neurodegenerative disorders. Driving the attentional competition within the right hemisphere between emotional stimuli and tasks that engage the right hemispheric networks to extreme levels may allow for novel insights into even subtle alterations in emotion–attention and emotion–cognition interactions that reflect the affective, cognitive and social challenges these clinical populations encounter. Furthermore, utilizing the competition for the right hemisphere’s limited pool of resources may provide sensitive biomarkers that reflect these alterations due to brain damage or disorder as well as their treatment. Different neuromodulation treatments would especially benefit from such biomarkers, allowing individualized optimization of treatment parameters based on the immediate impact of neuromodulation on the individual’s affective brain functions.

In addition to their potential for improving diagnosis of brain disorders and injury and allowing optimization of their treatments, EEG derived indices reflecting healthy right hemisphere-dependent emotion–attention interaction may also have potential in enhancing technological development towards optimal user experience. Furthermore, they may provide brain health indices that allow guiding wellness technologies and gearing work environments towards optimal brain health.

In conclusion, deeper understanding of normal and altered emotion–attention interaction in general and especially in the right hemisphere allow for developing novel biomarkers that reflect those interactions with wide scientific, clinical and technological impact and applicability.
